# A Survey of UK Public Interest in Internet-Based Personal Genome Testing

**DOI:** 10.1371/journal.pone.0013473

**Published:** 2010-10-19

**Authors:** Lynn F. Cherkas, Juliette M. Harris, Elana Levinson, Tim D. Spector, Barbara Prainsack

**Affiliations:** 1 Department of Twin Research and Genetic Epidemiology, King's College London, London, United Kingdom; 2 Division of Molecular Genetics, Department of Pediatrics, Columbia University Medical Center, New York, New York, United States of America; 3 Centre for Biomedicine and Society (CBAS), King's College London, London, United Kingdom; Yale University School of Medicine, United States of America

## Abstract

**Background:**

In view of the increasing availability of commercial internet-based Personal Genome Testing (PGT), this study aimed to explore the reasons why people would consider taking such a test and how they would use the genetic risk information provided.

**Methodology/Principal Findings:**

A self-completion questionnaire assessing public awareness and interest in PGT and motivational reasons for undergoing PGT was completed by 4,050 unselected adult volunteers from the UK-based TwinsUK register, aged 17 to 91 (response rate 62%). Only 13% of respondents were aware of the existence of PGT. After reading a brief summary about PGT, one in twenty participants (5%) were potentially interested at current prices (£250), however this proportion rose to half (50%) if the test was free of charge. Nearly all respondents who were interested in free PGT reported they would take the test to encourage them to adopt a healthier lifestyle if found to be at high genetic risk of a disease (93%). Around 4 in 5 respondents would have the test to convey genetic risk information to their children and a similar proportion felt that having a PGT would enable their doctor to monitor their health more closely. A TwinsUK research focus group also indicated that consumers would consult their GP to help interpret results of PGT.

**Conclusions/Significance:**

This hypothetical study suggests that increasing publicity and decreasing costs of PGT may lead to increased uptake, driven in part by the general public's desire to monitor and improve their health. Although the future extent of the clinical utility of PGT is currently unknown, it is crucial that consumers are well informed about the current limitations of PGT. Our results suggest that health professionals will inevitably be required to respond to individuals who have undergone PGT. This has implications for health service providers regarding both cost and time.

## Introduction

Since autumn 2007, commercial companies have been offering personal genomic testing (PGT) and disease risk calculations on the internet. For fees starting at just a few hundred dollars these companies look at more than a million variations (single nucleotide polymorphisms, SNPs) across the genome to assess their customers' individual genetic predispositions to various diseases (such as cardiovascular disease and diabetes), traits (like eye colour and ear wax), carrier status, and drug sensitivities.

PGT differs from clinical genetic testing in three main ways. First, instead of looking for the presence or absence of a single mutation, PGT identifies a large number of susceptibility loci for a wide variety of traits in order to calculate genetic risk. Second, PGT focuses on complex diseases and traits, which are caused by the interplay of various genetic and environmental factors, many of which are as yet unknown or unexplored; resulting in low predictive values [1]–[3]. An analysis of commercially available direct-to consumer PGT published in autumn 2009 showed that about 80% of reported relative risks were between 0.5 and 1.5. [4] Another important difference between clinical genetic testing and PGT is that consumers can purchase PGT without direct input from medical professionals. PGT can be purchased and results accessed directly through the internet [5]. This stands in sharp contrast to the high level of clinical care and counselling provided as part of a clinical genetic testing protocol.

In the future, PGT companies will either keep costs constant whilst improving the quality or scope of the information conveyed to the consumer [4] or PGT costs will decrease further, thus possibly encouraging PGT consumer uptake or facilitating the incorporation of elements of PGT into clinical use. Either way, it is likely that PGT will become more accessible and potentially more informative for a growing number of people. This will have tangible effects on a multitude of factors such as health care delivery and insurance [6]–[7]. To date, there has been no exploration in the UK of the factors that are likely to motivate individuals to consider PGT or the impact of PGT results on their lifestyle and life decisions. Due to the aforementioned differences between PGT and clinical genetic testing, findings from attitudinal studies in clinical genetics cannot be directly transposed to PGT.

To our knowledge this is the first survey of a large group of unselected individuals in the UK aiming to explore interest in PGT, the reasons people would consider taking such a test and how they intend to use the genetic risk information provided.

## Materials and Methods

### Ethics Statement

This study was conducted according to the principles expressed in the Declaration of Helsinki. The study was approved by the Institutional Review Board (Research Ethics Committee) of St Thomas' Hospital (approval no. EC04/015). All research subjects provided written informed consent for the collection of data and subsequent analysis.

### Study Population

Self-completion questionnaires were sent to 6,510 active adult twins aged from 16 years who are all volunteers on the TwinsUK Adult Twin Registry and have responded to at least one survey in the last four years. All were ascertained from the general population and shown to be comparable to age-matched population singletons. [8] These unselected monozygotic (MZ) and dizygotic (DZ) twins have been recruited since 1992 using twin registers and national media campaigns and have been used in a wide variety of studies (www.twinsuk.ac.uk). For historical reasons, the cohort is predominantly female with a mean age of 54 years (range 17–91years).

### Questionnaire

The PGT questions were based on issues raised in the Harvard University Personal Genetics Education Project [9] and questions included in the REVEAL study of Alzheimer's Disease. [10] These questions were included in an 8-page questionnaire covering a range of unrelated topics of clinical interest. Questionnaires were sent out in autumn 2008, almost exactly one year after the commencement of direct-to-consumer PGT and only a few months after one company (*23andMe*) cut the price to $399 (approximately £250), rendering the test more affordable for a broader range of people. Demographics such as age, gender, family structure and socio-economic status (SES) based on postcode were taken from either the current or earlier questionnaires. This information was used to create sub-groups in the analysis.

The following information was given as an introduction to the PGT-related questions:

“Since 2007 it has been possible to order a personal genetic screen over the internet. You send a sample of saliva to a commercial company who look at selected genetic markers. Based on these markers they estimate your personal lifetime genetic risk of developing around 20 common diseases (such as heart disease, Alzheimer's disease, glaucoma or diabetes). Results are sent via an email alert to a private web-link.”

Firstly, subjects were asked if they were aware of such PGT services. Then they were asked to indicate, on a 5-point Likert scale from *very likely* to *not at all likely* (where 1 = very likely, 2 =  fairly likely, 3 = undecided, 4 = not very likely and 5 =  not at all likely), how likely they would be to order such a test if the service cost £250. This question was then repeated with a scenario in which the PG test was available free-of-charge, to remove any financial considerations. All respondents who expressed at least some interest (i.e. codes 1,2 or 3) in taking a PG test if it was *free* were then asked to what extent they agreed or disagreed with a list of possible reasons why people may choose to take a personal genome test.

Responses were on a 5-point Likert scale from *strongly agree* to *strongly disagree* (where 1 = strongly agree, 2 =  tend to agree, 3 =  neither agree nor disagree, 4 = tend to disagree and 5 =  strongly disagree). Because the number of respondents expressing disagreement with any of the 5 statements was very low, the latter two codes (4 & 5) were combined for analytical purposes.

### Data Analysis

All analyses were performed using STATA 10 software. Respondents were divided into those under and over 50 years of age for comparison purposes, but Spearman rank correlations were used to assess the relationship between responses and actual age as well as between socio-economic groups. The chi-square test statistic was used to compare differences in responses between males and females and between those who had children and those who were childless. In addition, a test for trend was conducted to assess whether any observed trend between responses and the dichotomous variables is linear and therefore amenable to useful interpretation.

## Results

4,050 twins aged between 17 and 91 completed the questionnaire. This equals a response rate of 62% (Table 1). The mean age of respondents was 56 years, 89% were female and respondents lived all over the UK. Non-respondents were younger on average (mean age 50 years, range 17–91) and a slightly higher proportion were males than females (16% compared with 11% of respondents). The proportion of respondents in each of the socioeconomic status (SES) groups was a fair representation of the distribution within our cohort. Four in five respondents (79%) had children.

**Table 1:** Respondents characteristics.

		Number	%
AGE *	Under 50	1,144	28
	Over 50	2,906	72
GENDER	Female	3,624	89
	Male	426	11
CHILDREN #	Yes	2,935	79
	No	784	21
SES (IMD) #	1 (Lowest 20%)	247	7
	2	455	12
	3	749	20
	4	965	26
	5 (Highest 20%)	1264	35

*µ = 56.4; range 17-91;

# Total less than 4,050 due to missing responses.

### Interest in personal genetic testing (PGT)

The level of awareness of PGT was low with only 1 in 8 people (13%) having heard of the service (Table 2). Younger people were significantly more likely to be aware of PGT than older people, but there were no other demographic differences between those who were aware of PGT and those who were not. Level of interest in taking such a test was clearly dependent on cost. There was very little interest expressed in ordering a personal genome test if it cost £250; only 5% were very or fairly likely to order the test at this price, with those of lower SES expressing the least interest (p<0.01). However, with the scenario of PGT tests being available at no cost, interest rose significantly, with nearly half of all respondents (48%) saying they would be very or fairly likely to order such a test. A further 1 in 5 (22%) were undecided; with only 3 in 10 (30%) saying they were unlikely to order a test. Younger people and males expressed significantly more interest than older people and females (p<0.01). Respondents in the highest SES group were significantly less likely to order a personal genomic test if it was a free service compared with those in lower SES groups (p<0.02).

**Table 2:** Interest in Personal Genetic Screening.

	Total	AGE			GENDER			CHILDREN			SES (low–high)				
	4,050	<50	>50		F	M		Yes	No		1&2	3	4	5	
	%	%	%	P*	%	%	P	%	%	P	%	%	%	%	P*
**Awareness of Personal Genetic Screening tests**															
YES	13	15	13	**<**0.01	13	15	NS	13	14	NS	13	12	13	14	NS
NO	87	85	87		87	85		87	86		87	88	87	86	
**Likelihood of ordering test if £250**															
Very likely	2	1	2	NS	2	2	NS	2	2	NS	2	2	2	2	<0.01
Fairly likely	3	3	4		3	4		3	3		3	3	4	3	
Undecided	12	12	12		12	11		12	11		9	11	13	11	
Not very likely	33	34	33		33	39		34	32		31	31	33	37	
Not at all likely	50	51	50		50	44		49	52		55	53	49	47	
**Likelihood of ordering test if free**															
Very likely	30	35	28	**<**0.01	29	37	**<0.01**	30	29	NS	32	31	30	27	<0.02
Fairly likely	18	20	17		18	23		17	19		19	17	20	17	
Undecided	22	21	22		22	18		22	22		21	20	22	24	
Not very likely	16	14	17		17	12		16	17		14	18	15	18	
Not at all likely	14	10	16		14	11		15	13		14	14	13	15	

P, p-value from chi-square test for response differences between groups; bold  =  significant linear trend;

P*, p-value from spearman rank correlation of actual age/SES with response;

NS, not significant; % may not add up to 100 due to rounding.

### Reasons to undergo personal genomic testing

Attitudes to five putative reasons for taking a personal genomics test were analysed for those respondents who had expressed at least some interest in taking such a test if it was free (70% overall, N = 2,814) (Table 3).

**Table 3:** Reasons to take a Personal Genetic Screen.

	Total	AGE			GENDER			CHILDREN			SES (low–high)				
	2814	<50	>50		F	M		Yes	No		1&2	3	4	5	
	%	%	%	P*	%	%	P	%	%	P	%	%	%	%	P*
**Encourage me to adopt a healthier lifestyle if found to be at high genetic risk of a disease**															
Strongly Agree	55	57	55	<0.01	56	48	**<0.01**	54	59	NS	55	57	55	53	NS
Tend to Agree	38	36	38		37	41		40	33		38	36	38	40	
Neither Agree or Disagree	5	5	5		5	9		5	7		6	5	5	5	
Strongly/Tend to Disagree	2	1	2		1	3		2	1		1	1	1	2	
**Learn more about myself**															
Strongly Agree	47	51	46	<0.01	47	47	NS	46	50	NS	45	51	50	43	NS
Tend to Agree	39	36	40		39	40		40	38		41	36	37	43	
Neither Agree or Disagree	11	9	12		11	11		12	10		12	11	12	11	
Strongly/Tend to Disagree	3	3	2		3	2		3	2		2	2	2	3	
**Convey genetic risk information to my children**															
Strongly Agree	45	37	48	<0.01	46	33	**<0.01**	49	25	**<0.01**	44	50	45	40	NS
Tend to Agree	35	34	35		34	36		38	24		32	31	36	39	
Neither Agree or Disagree	15	20	13		14	22		11	34		18	14	13	15	
Strongly/Tend to Disagree	6	9	4		5	9		3	18		7	4	6	5	
**Doctor can monitor my health more closely**															
Strongly Agree	39	33	41	<0.01	40	31	**<0.01**	40	33	**<**0.01	40	40	38	36	NS
Tend to Agree	40	37	42		40	42		42	37		38	42	40	43	
Neither Agree or Disagree	16	22	14		16	19		15	20		17	13	18	17	
Strongly/Tend to Disagree	5	8	3		4	8		3	9		4	5	4	4	
**Assist in financial planning for the future**															
Strongly Agree	20	17	21	<0.01	20	15	**<0.02**	20	15	**<0.01**	19	21	20	18	NS
Tend to Agree	30	26	31		29	31		31	26		29	32	27	31	
Neither Agree or Disagree	35	37	34		35	33		35	35		38	32	39	33	
Strongly/Tend to Disagree	15	20	14		15	21		14	23		15	15	14	18	

(Base  =  very/fairly likely or undecided to order PG test if free);

P*, p-value from spearman rank correlation of actual age/SES with response;

P, p-value from chi-square test for response differences between groups; bold  =  significant linear trend;

NS, not significant; % may not add up to 100 due to rounding.

In this group, the most frequently endorsed reason for taking the test was to “encourage me to adopt a healthier lifestyle if found to be at high genetic risk of a disease”. More than 9 in 10 (93%) agreed with this statement, of whom 55% expressed strong agreement and 38% tended to agree. There was a significant correlation with age, with younger people more likely to endorse this reason than older respondents (p<0.01). Females were also significantly more likely to agree compared with males (p<0.01). Almost as many respondents (86% overall) agreed they would take a personal genomic test in order to “learn more about myself”. Again, the younger the respondents, the more likely they were to endorse this statement (p<0.01).

Approximately 4 in 5 respondents agreed that being able to “convey genetic risk information to my children” and so the “doctor can monitor my health more closely” were reasons for taking a personal genomic screen (80% and 79% respectively). Older respondents were significantly more likely to endorse both these statements than younger respondents (p<0.01), as were females compared with males (p<0.01) and those with children compared to those without children (87% and 49% respectively, p<0.01). Respondents were equally divided (50% agreed and 50% were undecided or disagreed) as to whether they would take a personal genome test in order to “assist in financial planning for the future” with only one in five (20% overall) expressing strong agreement with this statement. As could be expected, there was a correlation with age, with older respondents more likely to endorse this as a reason for taking the test (p<0.01). Females and those with children were also significantly more likely to endorse this statement than males and those without children (p<0.02 and p<0.01 respectively). Notably, we found no significant trends with SES and endorsement levels of any of the five listed statements.

## Discussion

Our findings suggest that at the end of 2008 awareness of PGT among the general public was still low (13%), but this may have increased over the past year with significantly more exposure in the media. Not surprisingly, cost is a decisive factor in the level of purported interest in taking such a test. The fact that only 1 in 20 (5%) expressed interest in PGT at a cost of £250 suggests uptake will not increase dramatically as long as costs remain stable. However, the high level of interest in a free personal genetic test (48% said they were very or fairly likely to order PGT and 22% were undecided) suggests that uptake may increase when costs decrease. Graves et al [11], in a telephone survey of 105 women with a family history of breast or ovarian cancer, also found that as costs increased interest in SNP-based risk testing decreased, but neither study assessed actual uptake. Although comparisons between countries should be treated with caution, similar hypothetical research in Russia [12] and among US users of the online social networking platform *Facebook* [13] reported similarly high levels of interest in PGT (68% and 64% respectively).

The US-based Multiplex Initiative assessed actual uptake rates of genetic susceptibility testing for eight common conditions in 2000 healthy individuals and found that after pre-test education, only 14% proceeded with testing [14]. Whereas we acknowledge that our ‘hypothetical uptake rate’ may indeed diminish in an actual testing scenario, crucial differences between the studies make comparisons complex. Subjects in the Multiplex Initiative belonged to US private health care organisation, were aged between 25 and 40, and had an educational session prior to deciding whether to undertake testing. In contrast, our subjects had a mean age of 56, were given only basic information on PGT (to mimic the non-clinical aspect of DTC internet testing) and are representative of members of the general public.

Sanderson et al [15] found that hypothetical interest only modestly predicted actual uptake of genetic testing for lung cancer risk. However, because the sample was small and highly selected and as participants were all smokers related to patients with lung cancer, results could generate high magnitudes of risk compared with the impact of results from PGT. Nonetheless, in this study, actual uptake was significantly higher among participants who said they “definitely would” take the test compared with others (45% vs 26%, p = 0.035). To our knowledge, neither hypothetical nor actual PGT uptake rates have been explored in the UK. We view a hypothetical study as a crucial first step towards assessing the public's interest in and attitudes to PGT and believe that our results warrant further research into actual uptake rates.

Our finding that younger people and males reported a higher level of interest in free PGT corresponds with findings from other studies on attitudes towards new health technologies, where older individuals [16] and females [17] were found to be more sceptical. Our results also suggest that for respondents in the highest SES group, the decision about whether or not to undertake PGT may be less influenced by cost.

Among those expressing at least some interest in PGT (N = 2,814), nearly all (93%) endorsed as a reason for taking the test to “encourage me to adopt a healthier lifestyle if found to be at high genetic risk of a disease”. Similarly, the aforementioned studies conducted in Russia and the US both reported that the vast majority of participants felt they would modify lifestyle if found to be at high risk for a complex disease [12], [13]. Numerous studies have investigated whether knowledge of genetic risk associated with one or several gene variants may motivate risk reducing behaviour - for conditions such as Alzheimer's disease, various cancers and obesity - due to the highly personalised nature of the information provided. However, they do not provide conclusive evidence of long-term behavioural change and clinical benefit [10]; [18]–[24]. In most cases, sample sizes have been small and the study population highly selected and non-representative of the general population. In the case of smoking cessation studies that disclose lung cancer susceptibility gene status to smokers (which represents the main basis for behavioural motivation research), this research is complicated by issues of addiction which may confound the motivational impact of genetic risk disclosure. Furthermore, as McBride *et al* [25] state in their comprehensive assessment of the behavioural response to personalised genetic risk profiles, it is likely that genetic risk messages are not always fully understood by recipients.

The motivational impact of DTC testing where personal genetic risk is based on numerous common genetic variants and is even more probabilistic has yet to be assessed in a realistic scenario. Hence our findings can be seen as an indicator of current intentions of the general public and their level of awareness of modifiable risk factors for many diseases. Further research is needed to assess whether disclosing personalised genetic risk for common complex diseases does indeed lead to clinically useful behavioural changes.

As many as four in five (80%) of those respondents in our survey who were interested in PGT endorsed as a reason for taking the test that their “doctor can monitor my health more closely”. Further in-depth discussion with a subset of these individuals in focus groups for a related research project (EL, MSc Thesis), clearly indicated that consumers felt they would need the help of their GP to interpret their results and to discuss subsequent implications for their future health. In the USA, McGuire [13] found a similar proportion of respondents (78%) would ask their physician to help interpret the results, even if the internet company that conducted the analysis provided help. Indeed, some PGT companies actively encourage customers to discuss their genetic test results with their physician, claiming that these results, together with their medical history, family history and lifestyle enable their doctor to take a more personal approach to monitoring their health. These findings could have serious implications for the UK National Health Services (NHS). Our results suggest that - even though the predictive value of these tests may be limited - there could be a considerable burden imposed on GPs, both in terms of time and costs, if uptake of PGT becomes more widespread in the future with patients requesting help from their GP to interpret their results.

This raises two important issues. Firstly, it is imperative that potential test-takers are educated as to the current limitations of PGT in terms of their predictive value (non-modifiable genetic risk) as well as the value of adopting a healthy lifestyle regardless of genetic susceptibility. Secondly, in addition to questioning whether interpretation of DTC PGT results should be the responsibility of GPs, the question arises as to their level of competency to explain results to their patients, as they are not currently trained to interpret the results of multi-factorial disease susceptibility tests and the clinical significance of genetic associations [26].

The UK House of Lord's Report on Genomic Medicine 2009 [27], the US Secretary's Advisory Committee on Genetics, Health and Society (SACGHS) Report on Genetics Education and Training [28], and other advisory bodies' recommendations on DTC genetic testing [29] have all recognised the need to train health professionals to adequately deal with patients seeking their help with interpreting PGT results and produce practice guidelines. Currently confidence among GPs in the UK regarding their expertise in genetics is reported to be low [27]. The clinical validity (predictive value of the test results) and the clinical utility (actionable outcomes and possibilities for prevention) of PGT is likely to increase in the coming decade. Hence, health professionals in many areas of health care will need access to up-to-date information pertaining to the scientific, ethical, regulatory, and societal dimensions of genetic susceptibility testing for complex diseases and conditions in order to guide their medical management.

Our study has several limitations. The first is the question of the generalisability of results from a twin-based cohort to the population as a whole. Due to their participation in genetic studies both identical and non-identical twins may be more aware of both the genetic and environmental contributions to disease than the general public. However, awareness of the availability of PGT was still low in this cohort (13%) and similar high intentions to modify lifestyle if found to be at high genetic risk of disease were reported in two studies of non-twins previously reported [12], [13]. Therefore attitudes to and expectations of PGT in our twins appear to reflect those of singletons.

Another potential limitation is that the demographics of the survey sample are not representative of the UK population as a whole as, for historical reasons, the sample is predominantly female (85%). Furthermore, as is the case for many volunteer study cohorts, there is a higher representation of the middle and higher social classes than the national average. Nonetheless, there were sufficient numbers in our study for statistically significant comparisons to be made between males and females and between four SES groups, as well as across ages. As the twin register was originally established as a resource for genetic research into diseases in Caucasian populations, this precluded the inclusion of individuals from ethnic minorities in this study. Further studies are warranted to investigate the attitudes of ethnic minorities towards PGT. However, this is further complicated by the fact that current PGT algorithms for risk may not be applicable to ethnic minorities due to differing genomic variation.

Finally, we acknowledge that the limited predictive value of current PGT for common complex diseases was not explained in detail to participants in our survey. However, the scenario presented reflects the limited level of information made available to consumers who currently take up DTC PGT over the internet and as such, the attitudes of our respondents are relevant. We have already stressed the importance of informing potential consumers of issues related to the clinical utility of genetic susceptibility testing. It remains to be seen whether uptake of PGT increases among the general public and whether it becomes a useful clinical tool for health professionals to improve public health. However, our findings suggest that health care systems must prepare for the potential ramifications of publicly-accessible genetic information on various aspects of health care delivery.
